# Shoulder instability surgery in Norway: The first report from a multicenter register, with 1-year follow-up

**DOI:** 10.3109/17453674.2011.641102

**Published:** 2012-04-24

**Authors:** Jesper Blomquist, Eirik Solheim, Sigurd Liavaag, Cecilie P Schroder, Birgitte Espehaug, Leif I Havelin

**Affiliations:** ^1^Department of Surgical Sciences, Faculty of Medicine and Dentistry, University of Bergen; ^2^Department of Surgery, Haraldsplass Deaconess Hospital; ^3^Department of Orthopedic Surgery, Sorlandet Hospital Arendal; ^4^Department of Orthopedic Surgery, Lovisenberg Deaconess Hospital; ^5^Norwegian Arthroplasty Register, Department of Orthopedic Surgery, Haukeland University Hospital, Norway; Correspondence: Jesper.Blomquist@kir.uib.no

## Abstract

**Background and purpose:**

In January 2008, we established the Norwegian Register for Shoulder Instability Surgery. We report on the establishment, the baseline data, and the results at 1-year follow-up.

**Methods:**

Primary and revision shoulder stabilization is reported by the surgeon on a 1-page paper form containing the patient's history of shoulder injury, clinical findings, and perioperative findings. The WOSI questionnaire for self-assessment of shoulder function is completed at baseline and at follow-up after 1, 2, and 5 years. To evaluate the completeness of registration, we compared our data with those in the Norwegian Patient Registry (NPR).

**Results:**

The NPR reported 39 hospitals performing shoulder stabilizations. 20 of these started to report to our register during 2009, and 464 procedures (404 primary, 59 revisions) were included up to December 31, 2009, which represented 54% of the procedures reported to NPR. Of the 404 primary procedures, 83% were operations due to anterior instability, 10% were operations due to posterior instability, and 7% were operations due to multidirectional instability. Arthroscopic soft tissue techniques were used in 88% of the patients treated for primary anterior instability and open coracoid transfer was used in 10% of such patients. At 1-year follow-up of 213 patients, we found a statistically significantly improved WOSI score in all types of instability. 10% of the patients treated with arthroscopic anterior labral repair and 16% treated with arthroscopic posterior labral repair reported recurrent instability. No statistically significant difference in functional improvement or rate of recurrence was found between these groups.

**Interpretation:**

The functional results are in accordance with those in previous studies. However, the incidence of recurrent instability 1 year after arthroscopic labral repair is higher than expected.

In Scandinavia, national quality registers have been used for many years to monitor outcome, thus making it possible to pinpoint inferior treatment methods (Herberts and Malchau 2000, Irgens 2000, Pahlman et al. 2005).

There are nationwide registers for joint replacement in several countries, and cruciate ligament registers have been established in Sweden, Denmark, and Norway (Granan et al. 2009). Most orthopedic registers are based on a simple reporting system whereby the surgeon completes a registration form, which is transferred to a secretariat either electronically or by post. In the Scandinavian cruciate ligament and hip fracture registers, the patients are also asked to complete an outcome questionnaire (Gjertsen et al. 2008, Granan et al. 2009). The reporting systems are simple to use and fast to fill in, and in return the hospitals are provided with feedback on the outcome of their patients, which are easily compared to that of the average outcome. The simplicity of use and the feedback provided are of benefit to each hospital, which may explain the high compliance rate (Espehaug et al. 2006).

To our knowledge, no national quality registers for shoulder instability surgery have been reported yet (Pulavarti et al. 2009). The experience from other orthopedic registers and the reported disparity concerning the results of surgery for shoulder instability was the background for establishment of a shoulder instability register. During the last decade, arthroscopic stabilization techniques have replaced open surgery to a large extent, as in some studies the short- and medium-term results of the former techniques have been found to be similar to those of open Bankart repair (Sperber et al. 2001, Bottoni et al. 2006, Fabbriciani et al. 2004). However, some other studies with medium-term or long-term follow-up have shown less satisfactory results after arthroscopic Bankart repair, with recurrent instability in 15.3–23% of patients (Boileau et al. 2006, Castagna et al. 2010). Thus, non-anatomical methods such as the Latarjet procedure have been proposed for use in patients with concomitant risk factors of recurrence (Boileau et al. 2006, Burkhart et al. 2007).

The aims of our recently established Register for Shoulder Instability Surgery are to collect epidemiological data, to evaluate the results of different treatment methods, to identify prognostic factors associated with good and poor outcome, and to facilitate improved treatment through direct feedback to the participating hospitals. In this article we describe the register, the methods used, the baseline data of the patients included, and our experience during the first 2 years of operation of the register. We also give the preliminary results of shoulder instability surgery, based on 1-year follow-up data.

## Patients and methods

Based on the experience from a pilot study performed in 2006 at 12 hospitals and involving 107 patients (Liavaag et al. 2007), a working group was set up to plan the establishment of a Norwegian shoulder instability register. The first registration started in January 2008. 39 hospitals performing shoulder stabilization surgery were identified in the Norwegian Patient Register (NPR; www.npr.no) and they were invited to participate in the register.

Eligible for inclusion in the register were Norwegian-speaking residents of Norway undergoing primary or revision surgery for shoulder instability. All directions of instability and both dislocation and subluxation are accepted for inclusion in the register. The patients are asked to complete the Western Ontario Shoulder Instability Score (WOSI) (Kirkley et al. 1998) and to provide information about their profession and their level of sports activity.

The WOSI score consists of 21 items divided into 4 domains, to be answered using visual analog scales. The total score is presented as a number between 0 (best) and 2,100 (worst), or transformed to a score where 100% equals normal shoulder function and 0% is the worst possible outcome. We use a Norwegian version of the WOSI score that has been translated and validated according to the guidelines presented by Guillemin et al. (1993). At 1, 2, and 5 years after the primary or revision surgery, the patients are asked to complete the same questionnaire. Furthermore, they are asked if they have experienced any new episodes of shoulder dislocation or if they have had additional surgery in the same shoulder. If additional surgery has been performed to the same shoulder, consent is obtained to retrieve hospital records regarding the surgery.

Based on the experience of the pilot study, a registration form was designed (Appendix 1). The aims were to define the type of instability, to describe the surgical treatment, and to identify patient characteristics that might influence the risk of recurrent instability and functional outcome. Previous shoulder surgery, pathology in the opposite shoulder, history of injury, direction and degree of instability, duration of symptoms, minimum activity level to trigger instability symptoms, and number of dislocations were recorded. Any glenoid bone defects and injuries to the labrum and/or capsule were marked on a schematic drawing of the shoulder. Humeral head defects, tendon injuries, or other findings were recorded. The surgical procedures were described schematically, including descriptions of the types of implants used and their positioning. To obtain correct information for the implants used, the surgeons were encouraged to provide the identification stickers supplied by the manufacturer.

Revision stabilization is defined as the hard endpoint of the register, whereas soft endpoints are patient-reported recurrences of instability and WOSI score at follow-up.

To estimate the national coverage of the register, the data were compared to those in the Norwegian Patient Register (NPR). The NPR contains a modified NOMESCO Classification of Surgical Procedures (NOMESCO 2009) for all procedures performed in public or private hospitals that are funded by the Norwegian public social security. The NPR contains information on surgical procedures performed and patient age, gender, and co-morbidity, but no information regarding outcome.

### Statistics

Mean substitution was used for replacing missing data. Distribution, with floor and ceiling effects, were analyzed. Subscores of 0–1% or 99–100% were considered to be extreme values, representing a floor or ceiling effect. Mean changes in WOSI score when comparing preoperative and 1-year results are given with 95% confidence intervals (CIs), and they were evaluated with paired t-test. Rates of recurrence in the different groups were compared with the chi-square test. Values of p < 0.05 were considered to be statistically significant. SPSS statistical software version 18.0 was used for the analyses.

### Ethics

The register was designed to comply with the ethical standards of the revised Helsinki Declaration of 2000. Participation is voluntary, and confidentiality is ensured for the patient and for the surgeons. The patients are informed about the aim of the study and about the kinds of data that are collected. They are also informed that they may withdraw from the register and have their personal data deleted at any time. The register was approved by the Regional Ethics Committee (Rek-vest 245.07). The collection and storage of data was approved by the Norwegian Data Inspectorate (NSD 1791).

## Results

The registration started in 8 hospitals in January, 2008. During 2008, 10 more hospitals joined the register, and 2 more started registration in the spring of 2009. During the period from January 2008 to December 2009, 464 stabilization procedures were recorded (Table 1). No open labral repair or arthroscopic bony procedures were recorded.

**Table 1.** Distribution of surgical procedures

	Primary	Revision	Total
*Anterior stabilization*			
Arthroscopy			
Bankart	294	25	322
Capsular plication	6	3	6
Sum	300	28	329
Open			
Latarjet	35	26	60
Capsular shift	1	1	2
Sum	36	27	62
Total	336	55	391
*Posterior stabilization*			
Arthroscopy			
Bankart	31	1	32
Capsular plication	8	0	8
Sum	39	1	40
Open			
Bony	0	0	0
Capsular shift	1	0	1
Sum	1	0	1
Total	40	1	41
*Multidirectional stabilization*			
Arthroscopy			
Bankart	14	1	15
Capsular plication	14 **^a^**	1	15
Sum	28	2	30
Open			
Bony	0	0	0
Capsular shift	0	1	1
Sum	0	1	1
Total	28	3	31

**^a^** 3 with rotator interval closure

### Patient characteristics

89% of the primary anterior dislocations had a traumatic debut. 60% of the injuries were related to sports. Winter sports (20%) and ball games (17%)—including soccer and team handball—were the most common causative activities. 18% of cases were caused by a fall during daily non-athletic activities and 7% were caused by road traffic accidents, including bicycling (Table 2).

**Table 2.** Patient characteristics for primary stabilization

	Anterior	Posterior	Multidirectional
	(n = 336)	(n = 40)	(n = 28)
Sex (%male)	68	73	61
Age (median, range)	25 (13–74)	28 (14–56)	25 (10–45)
Instability in contralateral shoulder, n (%)			
	34 (10)	5	12
Traumatic debut, n (%)			
definitive	299 (89)	20	11
uncertain	20 (6)	9	4
Month of symptoms, median (range)	28 (0–489)	34 (4–234)	60
Most common activities at injury (n)	Daily activity (55)	Daily activity (6)	Soccer (2)
	Ski (43)	Handball (5)	Ski (2)
	Soccer (24)		
	Handball (20)		
	Snowboard (17)		
	Epilepsy (10)		
	Assault (10)		
	MC (8)		
	Volleyball (8)		
	Car (7)		
	Weight lifting (7)		
Prophylactic antibiotics, n (%)	223 (69)	27	17
NSAID, n (%)	97 (31)	11	9
Day surgery, n (%)	133 (41)	8	9
Surgery position (beach), n (%) **^a^**	86 (30)	3	3
Operating time (median, range)	70 (18–200)	86	84
Concomitant SLAP lesion, n (%) **^a^**	70 (23)	12	6
Glenoid fracture, n (%)	21 (6)	0	0
Glenoid resorption, n (%)	28 (8)	1	0
HAGL (anterior or posterior)	0	1	1
Number of suture anchors (mean) **^a^**	2.61	2.38	2.39

**^a^** in arthroscopic procedures

### Preoperative functional score

Of the 464 patients who were included, 435 (94%) completed the preoperative WOSI form. Of these 435 WOSI forms containing a total of 9,135 items, data were missing for 85 items (0.9%) (Table 3). We found a normal distribution for the total score and all subscores, except for the emotional subscore for primary and revision anterior stabilization. In these groups, we found a positive skew, with a floor effect of 3% for primary cases and 8% for revision cases.

**Table 3.** Preoperative WOSI, percent of maximum score: mean (SD)

	Primary anterior	Revision anterior	Primary posterior	Primary multidirectional
	(n=314)	(n=50)	(n=39)	. (n=28)
WOSI total score	51 (18)	44 (18)	45 (14)	42 (16)
Physical symptoms and pain	59 (20)	52 (21)	48 (14)	45 (20)
Sport, recreation and work	40 (21)	34 (20)	41 (19)	38 (19)
Lifestyle and social functions	53 (23)	44 (23)	51 (21)	46 (20)
Emotions	36 (24)	30 (24)	36 (21)	31 (21)

### One-year follow-up

213 of 296 patients who were included until June 2009 completed the follow-up WOSI index and 210 also reported on recurrence of instability and additional surgery (Table 4). For all groups with primary stabilization and with anterior revision stabilization, we found a statistically significant improvement in shoulder function after 1 year. 10% of the patients who were treated with arthroscopic anterior labral repair and 16% of those treated with arthroscopic posterior labral repair reported having experienced at least 1 recurrent dislocation at the time of follow-up. There were no statistically significant differences between the groups regarding change in WOSI score, rate of recurrence, or rate of reoperation.

**Table 4.** Results at 1-year follow up after stabilization procedures

	Arthroscopy primary anterior	Arthroscopy revision anterior	Open primary anterior	Open revision anterior	Arthroscopy primary posterior	Arthroscopy primary multidirectional
	(n=141**^a^**)	(n=13)	(n=10)	(n=10)	(n=25)	(n=13)
Preop WOSI%, mean (SD)	51 (19)	48 (16)	55 (17)	48 (18)	45 (15)	42 (19)
1-year WOSI%, mean (SD)	75 (19)	64 (18)	80 (16)	69 (18)	63 (28)	76 (19)
Change in WOSI%, mean (CI 95%)	24 (20–27)	16 (3–30)	26 (13–38)	21 (14–28)	17 (8–26)	34 (24–45)
p-value	p<0.001	p=0.02	p=0.001	p<0.001	p=0.001	p<0.001
Recurrent instability(n)	14	0	0	0	4	0
Reoperation (n)	6	0	1	0	2	1

**^a^** n=138 for rate of recurrence and reoperation

### National coverage of the register

In 2009, 315 stabilization procedures were included in the shoulder instability register. 587 patients were entered into the Norwegian Patient Register during the same period.

## Discussion

With 4.8 million residents in Norway and 587 procedures (according to the NPR), the annual incidence of shoulder stabilization surgery in 2009 was 12 per 105 inhabitants. The male-to-female ratio for surgery was 2.2 to 1. The corresponding figure for Sweden, when the NOMESCO classification is used, was the same, with a 2.9-times higher rate for men than for women (Socialstyrelsen 2008). The annual number of procedures coded as shoulder stabilization in the NPR increased from 486 in 2007 to 587 in 2009. The trend was the same in Sweden, with an increased incidence of shoulder stabilization of 37% between 2006 and 2008 (Socialstyrelsen 2008). We have not found any other incidence figures regarding instability surgery in other countries.

Based on the information provided by the NPR, we estimate that about 54% of all stabilization procedures performed in 2009 were included in the register. For the Norwegian Hip Fracture Register, the registration completeness after 2 years of operation was 79% (Gjertsen et al. 2008). Compliance rates for the Nordic cruciate registers after the start-up phase were reported to be 85% for Denmark, 97% for Norway, and approximately 70% for Sweden (Granan et al. 2009). For the Norwegian Arthroplasty Register, the registration completeness is 97% (Espehaug et al. 2006). However, the completeness and accuracy of the NPR data are debatable, as the NOMESCO classification—which is used in the NPR—has no specific codes for different stabilization techniques, revision surgery, or SLAP repair. Thus, SLAP repairs might have been reported to the NPR as stabilizations, while some stabilization procedures might have been registered under other codes. Validation studies of both NPR data and shoulder register data are warranted to determine the true incidence of shoulder stabilization surgery in Norway. The shoulder instability register was started through a network of shoulder surgeons and not by the Norwegian Orthopedic Association, as were the other registers, which might explain a lower rate of hospital recruitment. A higher proportion of eligible procedures are performed at private day surgery units for this register than for the other orthopedic registers. For other orthopedic registers in Norway, the completeness rate increased over time and we believe that the same will be the case for this register.

Of the 404 primary procedures registered, 83% of the patients had anterior instability, 10% had posterior instability, and 7% had multidirectional instability. The distribution of type of procedures in our register is in accordance with the incidence of shoulder instability reported by others. For example, Owens et al. (2007) found that anterior instability comprised 80% of the instability cases. For primary anterior stabilization, arthroscopic soft tissue techniques predominated and were performed in 88% of the cases, while a coracoid transfer procedure was performed in 10% of cases. For MDI and posterior instability, only soft tissue techniques were used. We have not found any population-based studies in the literature that describe the frequency of the different techniques used.

The functional results as expressed by the WOSI score for arthroscopic anterior stabilization are in accordance with those in other studies (Bottoni et al. 2006, Mologne et al. 2007). The WOSI score is less used than the rating sheet for Bankart repair presented by Rowe et al. (1978). Direct comparison with many other studies is therefore difficult. The WOSI score was considered to be the most appropriate functional outcome score for the register, as it is validated, internationally acknowledged, and can be administered by post. Validation studies have shown a high effect size, allowing detection of clinical change in individual patients and groups (Salomonsson et al. 2009). Clinician-based outcome measures have historically had widespread use, but the use of patient-reported outcomes has increased. The need for physical examination excluded the use of the Rowe score in our register study.

In our material, we had a patient-reported recurrence incidence 1 year after surgery of 10%, in comparison with a figure of 15.3% 3 years postoperatively reported by Boileau et al. (2006) and 23% reported by Castagna et al. (2010). These articles describe an even rate of recurrence episodes during the follow-up period. We must therefore expect a higher degree of recurrence in our material over time. Of 463 procedures recorded in the register, 58 were revisions (13%). We need to follow the patients over a longer time period before we can make any conclusions about the true revision and re-revision rates.

The main aim of the register is to identify prognostic factors for the clinical outcome after surgery, by way of patient characteristics, perioperative findings, and procedures performed. The selection of items for the registration form was based on literature review, clinical experience, and experience from the pilot study. The register is for presently underpowered for analysis of prognostic factors, and further data collection is needed to answer such questions.

In summary, the Norwegian shoulder surgeons have opted to use modern arthroscopic techniques. No arthroscopic bony techniques and very few open soft tissue stabilizations are reported, while open coracoid transfers are performed regularly. The functional result is in accordance with previous studies. We found that the incidence of recurrent instability after arthroscopic labral repair was higher than expected one year after surgery. Longer follow-up and larger numbers of patients are needed before we can assess the effects of different prognostic factors on the results.
